# Sex differences in risk of developing Type 2 Diabetes Mellitus (T2DM): A feasibility assessment of FINDRISC scoring and barriers to disease management in a low-income settlement of Rawalpindi, Pakistan

**DOI:** 10.1371/journal.pgph.0003087

**Published:** 2025-07-16

**Authors:** Taimoor Ahmad, Aisha Tauqir, Heena Tariq, Asra Qureshi, Shirin Jalaluddin, Unab I. Khan, Naaznin Lokhandwala, Adnan Ahmad Khan, Ayesha Khan

**Affiliations:** 1 Akhter Hameed Khan Foundation (AHKF), Islamabad, Pakistan; 2 Research and Development Solutions (RADS), Islamabad, Pakistan; 3 Department of Family Medicine, The Aga Khan University, Karachi, Pakistan; 4 Glucose Trail; PLOS: Public Library of Science, UNITED STATES OF AMERICA

## Abstract

Type 2 Diabetes Mellitus (T2DM) is a growing global public health concern, especially in Pakistan where an estimated 33 million people (aged 20–79 years) have diabetes. This pilot study explores T2DM risk using FINDRISC scoring, and examines participants’ knowledge, attitudes, and costs of care related to T2DM across sexes.Adult participants (aged 25–65 years) residing in a low-income neighborhood in Rawalpindi, Pakistan were selected using multi-stage cluster sampling. Data was collected via SurveyCTO® software-based questionnaire, incorporating the standardized FINDRISC tool and questions assessing diabetes-related knowledge, management practices, and the costs associated with disease management. Standardized anthropometric measurements were obtained for all individuals. Descriptive analysis, cross tabulations by sexes, Chi-square, and Fisher Exact probability tests were performed using Stata 17. The study included 260 participants (84 men, 176 women) with a mean age of 41 ± 11.8 years. Anthropometric measures revealed obesity in 44% and elevated waist circumference in 45% of men and 83% of women. Based on FINDRISC scoring, 9 (13%) men and 30 (30%) women exhibited a high risk of developing T2DM (p < 0.01). Despite demonstrating higher knowledge and positive self-management practices towards diabetes, a higher proportion of women were classified as high and very high risk of developing T2DM compared to men (30% vs 13%, p < 0.01). Among self-reported cases of T2DM (49 participants), only 63% reported paying for their treatment, with women reporting higher average monthly expenditures than men, though differences were not statistically significant. Hence, despite higher knowledge and positive self-management practice toward diabetes, women are at greater risk of developing T2DM. The findings suggest the need for expanded community testing using the FINDRISC tool T2DM risk assessment in low-income settings and linking high-risk individuals to healthcare providers. Additionally, targeted health awareness campaigns among poor urban residents, particularly addressing socio-cultural barriers that increase T2DM risk among women, are recommended.

## Introduction

T2DM is a significant public health concern globally. An estimated 422 million people live with diabetes worldwide, with its prevalence increasing over time. Though the probability of dying from primary non-communicable diseases such as cardiovascular diseases, cancer, and chronic respiratory diseases has decreased globally by 22% between 2000–2019, the mortality rate for diabetes has increased by 13% [1,2]. By 2030, T2DM is expected to become the seventh leading cause of death globally.

Studies between 2019 and 2022 indicate that diabetes prevalence ranges from 17% to 26% in Pakistan, with variations attributed to different assessment methods, including self-reporting, clinical diagnoses, and screening tests. Based on these prevalence rates, Pakistan has an estimated 33 million people with diabetes aged 20–79 years, placing it 3^rd^ among countries with the highest diabetes burden globally [3–5].

Type 2 diabetes mellitus (T2DM) is the most prevalent form and accounts for more than 90% of all cases. Risk factors for T2DM include a genetic pre-disposition, physical inactivity, obesity, tobacco & substance use, and unhealthy eating habits [2]. Over time, T2DM leads to a myriad of complications and comorbidities such as increased incidence of cardiovascular disease, renal disease, and mortality that, in turn, result in absenteeism, disability, reduced quality of life, and premature mortality [6]. In a representative sample of the Pakistani population, it was found that 93.7% of individuals with Type 2 Diabetes Mellitus (T2DM) presented with comorbid health conditions [7]. These conditions were notably more prevalent among older patients, men, and urban residents [7]. The most common comorbidities identified included chronic kidney disease (15.8%), generalized anxiety disorder (14.4%), bone diseases (14%), and cardiovascular disease (12.6%) [8]. Such findings underscore the importance of effective education on diabetes, emphasizing both its prevention and management, as many of these complications and comorbidities are preventable.

However, the ground realities advocated by several global and national studies suggest that inadequate disease awareness, misconceptions, and cultural beliefs contribute to poor adherence to self-care and hinder diabetes control [9–12]. These are exacerbated by social, economic, and psychological factors such as depression, the lack of access to treatment, and affordability of healthcare services [13–15].

A significant challenge in Pakistan, as well as many other developing countries, is the lack of comprehensive and reliable data regarding non-communicable diseases (NCD), particularly diabetes, within the community. Existing national surveys in the country, such as the Pakistan Social and Living Standards Measurements Survey (PSLM), Pakistan Demographic Health Survey (DHS) and Multiple Indicator Cluster Survey (MICS), primarily emphasize areas such as children’s immunization, reproductive health, and infectious diseases, including diarrhea, malaria, and HIV with little data captured on NCDs [16]. This absence of NCDs or diabetes-related data severely hampers both the public and private sectors’ ability to effectively target and implement prevention and treatment strategies [17].

Population-level diabetes screening through existing health surveys is limited by the need for specialized diagnostic testing and laboratory infrastructure. Given these resource constraints, our study assessed: (1) the feasibility of implementing the FINDRISC tool as a screening method in an urban poor settlement in Pakistan, (2) community knowledge and self-management practices regarding diabetes, and (3) healthcare costs among those with diagnosed diabetes. This approach aimed to explore screening options that could potentially complement traditional diagnostic methods in resource-limited contexts.

The FINDRISC predictive tool was developed by Lindstrom and Tuomilehto [18], and has been shown to have high sensitivity and specificity in identifying diabetes risk within South Asian populations as compared to alternative methods [19–21].

## Method

### Ethical statement

The study protocol was reviewed and approved by the Research and Development Solutions (RADS) Institutional Review Board (IRB), which is registered with the Office for Human Research Protection (OHRP) (Reference Number: IRB00010843). Each participant was assigned a unique study identifier (Study ID) to maintain participants’ confidentiality. Oral informed consent was obtained from all participants (adults aged 25–65 years). Written consent could not be obtained due to literacy barriers among participants in the low-income community setting and concerns about creating additional barriers to participation. The RADS IRB approved the use of oral consent for this study. Oral consent was documented electronically through the SurveyCTO® platform, where the consent statement was read aloud by trained enumerators and participants’ responses were recorded as yes/no before proceeding with the survey. Participants were explicitly informed about voluntary participation, their right to withdraw at any time without consequence, and that non-participation would not result in any harm or loss of community networking with the RADS team.

Participants were categorized by biological sex (male/female) based on self-identification, as sex-specific risk factors for Type 2 Diabetes Mellitus are well-established in the literature and relevant to the study’s clinical objectives. Age was categorized into standard epidemiological groups within the 25–65 years range, aligning with established diabetes risk assessment protocols and the target demographic for FINDRISC scoring. Additional demographic categorizations included marital status, ethnicity, educational attainment, occupation, and household income levels. These categorizations were based on participants’ self-reported information and followed standard demographic classification systems used in Pakistani population studies. The inclusion of these variables was justified by their established associations with diabetes risk factors and health-seeking behaviors in South Asian populations. In the analysis, we controlled for potential confounding effects of age group, marital status, ethnicity, education, occupation, and income to isolate the independent effects of sex on diabetes risk and health behaviors. The selection of participants from the low-income neighborhood of Dhok Hassu, Rawalpindi was justified by the need to assess diabetes risk in underserved populations where healthcare resources are limited and diabetes burden is disproportionately high.

### Study design

This is a descriptive, cross-sectional study conducted sequentially in two phases. The first phase was conducted from 30^th^ June – 9^th^ August 2021, followed by the second phase conducted from 6 – 9^th^ September 2021 in the same location of Dhok Hassu, Rawalpindi.

### Study setting and population

The study was conducted at a sentinel surveillance site operated by the Akhter Hameed Khan Foundation (AHKF) and Research and Development Solutions (RADS) since 2015. Dhok Hassu is a low-income urban community in Rawalpindi, Pakistan, with a population of 278,000. The community is characterized by a nominal median daily income below USD 1.90 (USD 7.5 purchasing power parity) per person. Educational attainment among residents shows women typically reaching elementary school level and men achieving primary school level. 75% of men work as day laborer or are self-employed, with a significant portion employed at the adjacent vegetable market. Women’s employment outside the home is minimal (<3%). Mobile phone access shows a marked gender disparity, with 87% of men having access compared to 35% of women.

### Sample size and sampling

The sample size was calculated using UNICEF Multiple Indicator Cluster Survey (MICS) 3 sampling tool, developed in 2005 to determine an appropriate sample size that ensures statistically valid, cost-efficient and representative survey results [22]. For the present study, the calculator was set at a design effect of 2.5, with a relative margin of error at 5%, a confidence interval at 95% and a response rate of 90%. The yielded sample size of 385 was rounded up to 400 participants.

We implemented a cluster randomization sampling design, with clusters proportionally distributed across neighborhoods based on population density. Household selection followed a right-hand rule methodology, sampling every third household. In cases of refusal, the adjacent household was approached. Households sharing a common cooking stove were considered a single unit [23]. One adult participant (aged 25–65 years) was selected from each household based on availability and willingness to participate at the time of the survey visit.

### Participants’ selection and study teams

During the first phase, participants were identified and recruited for the study after obtaining verbal consent. Those who consented were interviewed by the data collectors with interviews administered digitally using SurveyCTO® software. During the second phase, the participants were re-approached for anthropometric (height, weight, and waist circumference) measurements. Height was measured without shoes using scale ‘Seca 213’ and rounded to the nearest millimeter. Weight was recorded using a ‘Seca 813’ digital floor weight scale placed on a firm and flat surface and rounded to the nearest 100 grams. Waist circumference was measured above the navel using a flexible, non-stretchable tape, ensuring no compression of the skin, and recorded to the nearest 0.1 cm. Trained data collectors collected data on these measurements organized into teams of one male and one female interviewer. These data collectors received formal training from the investigative team, covering study participant selection, data collection protocols, quantitative interview techniques, standardized procedures for anthropometric measurements, and the use of the SurveyCTO® software.

### Survey questionnaire

The questionnaire was developed in English and translated into Urdu (local language). The translated questionnaire underwent an in-house pilot test involving 10 participants prior to training data collectors and the field study team. This pilot phase aimed to ensure that the questions’ contextual integrity and intended meaning were accurately preserved in the translated version. The tool captured information on socio-demographic characteristics, anthropometric measures, T2DM risk screening using FINDRISC scoring, medical history of chronic diseases, and knowledge and self-management behaviors regarding diabetes along with cost of treatment.

The FINDRISC predictive tool, developed by Lindstrom and Tuomilehto [18], was employed to measure diabetes risk in adults. The description and the coding of the variables used in the study are presented in S1 Table. The tool consists of eight self-reported variables: age, body mass index (BMI), waist circumference, daily vegetables and fruit intake, family history of diabetes, history of high blood pressure, and hyperglycemia. The tool generates scores ranging from 0-26, with higher scores indicating increased diabetes risk. While the original FINDRISC tool uses self-reported anthropometric measures, we opted to calculate BMI and measure waist circumference directly using standardized procedures and equipment, as described in the data collection section. This approach was essential in our low literacy setting, where concepts such as BMI and specific waist circumference measurements would be unfamiliar to many participants.

The knowledge and attitude questions regarding disease management were partially adapted from several standardized and validated tools, including the Michigan Diabetes Research and Training Center’s Diabetes Knowledge Test (DKT) [24], Diabetes Knowledge Questionnaire (DKQ) [25], Diabetes Risk Score Assessment tool and Summary of Diabetes Self-Care Activities (SDSCA) [18,25].

### Statistical analyses

Descriptive analyses of socio-demographic and clinical characteristics were produced. Mean ± Standard Deviation was reported for normally distributed continuous variables, and median ± IQR was computed for non-normally distributed variables. FINDRISC scale risk categorization was analyzed using standard scoring classification, with associations to socio-demographic variables assessed through chi-square tests. Knowledge and self-management behavior were evaluated using a 3-point Likert scale (agree; neutral; disagree). Sex-based associations with knowledge and self-management practices were analyzed using chi-square or Fisher’s exact tests. Diabetes management costs of participants with diabetes in the survey (n:49) were analyzed descriptively. All analyses were performed using Stata/SE17.0®.

## Results

**Fig 1:**
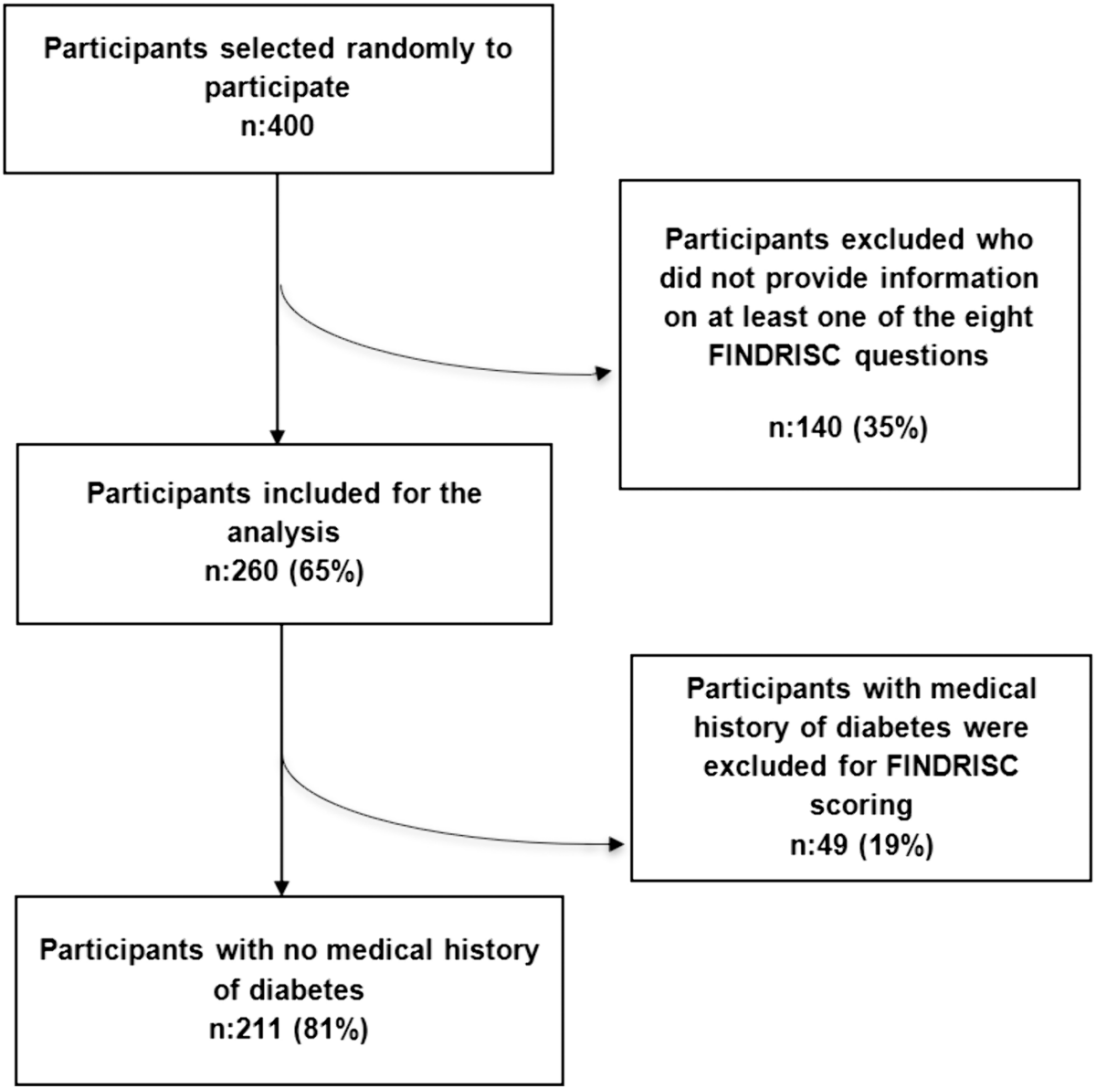
Flow chart of study sample.

Fig 1 illustrates phase-wise participant selection. Of the 400 initial participants who participated, 140 (35%) were excluded due to incomplete responses on at least one of the eight FINDRISC questions. The final analysis, therefore, included 260 respondents. For the FINDRISC analysis specifically, we further excluded 49 (19%) participants who had a confirmed clinical diagnosis of diabetes.

### Socio-demographic and clinical details

Women comprised a significantly larger proportion of participants than men (67% vs. 32%, Χ^2^:32.5; *p*- < 0.01). The average household size was 9.0 ± 4.9 members, with 81% of the participants being native residents of Dhok Hassu. Ethnically, 41% of participants were Punjabi-speaking. Educational attainment was notably low, with 44% (n = 116) having no formal education. Regarding occupation, 12% of male participants were daily wage earners, while 87% of female participants were housewives. Income data revealed that 107 (41%) participants reported an average monthly income of PKR 15,500 (USD 53.90) (Table 1).

**Table 1:** Socio-demographic and clinical details of study participants (N = 260).

(A) Sociodemographic details				
Characteristics	Total n(%)	Men n(%) 84(32)	Women n(%) 176(68)	p-value
**Age in years**				
Mean ±SD	40.8±11.8	40.4 ±11.8	41.0 ±11.6	0.73^#^
**Marital Status**				
Single	20(7.6)	13(15)	7(4)	0.002^^^^
Married	235(90)	71(84)	164(93)	
Widowed	5(2)	-	5(3)	
**Ethnicity**				
Urdu	26(10)	5(6)	21(12)	0.06^^^^
Punjabi	107(41)	30(35)	77(43)	
Pushto	101(38)	38(45)	63(36)	
Hindko	16(6)	10(12)	6(3.4)	
^!^ Others	10(4)	1(1.1)	9(5.1)	
**Education**				
No formal education	116(44)	24(28)	92(52)	0.009^^^^
Primary education	38(14)	14(16)	24(13)	
**Middle education	35(13)	14(16)	21(12)	
*Secondary education	41(16)	18(21)	23(13)	
Intermediate education	18(7)	10(12)	8(4.5)	
***Higher education	12(5)	4(4.7)	8(4.5)	
**Occupation**				
Unemployed	14(5)	14(16)	-	<0.01^^^^
Daily wager	32(12)	22(26)	10(5.6)	
Own business (shop etc.)	24(9)	23(27)	1(0.5)	
Teacher	3(32)	1(1.1)	2(1.1)	
Housewife^+^	154(87)	-	154(87)	
Health care provider (nurse, dispenser)	2(0.7)	1(1.1)	1(0.5)	
Others^++^	31(12)	23(27)	8(4.5)	
**Household monthly income (PKR)**				
<11000 PKR	9(3.4)	3(3.5)	6(3.4)	<0.01^^^^
11000-20000PKR	107(41)	17(20)	90(51)	
21000-30000PKR	82(31)	27(32)	55(31)	
31000-40000PKR	23(9)	11(13)	12(7)	
41000-50000PKR	30(11)	19(22)	11(6.2)	
>50000PKR	9(3.4)	7(8.3)	2(1.1)	
**Household monthly expenditure (PKR)**				
<11000 PKR	8(3)	3(3.5)	5(3)	<0.01^^^^
11000-20000PKR	99(38)	15(18)	84(47)	
21000-30000PKR	80(30)	31(37)	49(28)	
31000-40000PKR	46(17)	20(24)	26(14)	
41000-50000PKR	18(7)	10(12)	8(4.5)	
>50000PKR	9(3.4)	5(5.9)	4(2.2)	
**(B) Anthropometrics and clinical details**				
**BMI (Kg/m2)**				
Underweight (BMI<18Kg/m2)	8(3)	5(6)	3(1.7)	0.02^^^^
Normal weight (BMI>=18 & <=22.9Kg/m2)	42(16)	12(14)	30(17)	
Overweight (BMI>=23 & <=26.9Kg/m2)	95(36)	22(26)	73(41)	
Obese (BMI>=27Kg/m2)	114(44)	44(53)	70(39)	
**Waist circumference**				
Median± IQR	91.4(81.2-96.5)	87.6(81.2- 96.5)	91.4(71.1- 121.9)	0.14^^^
Medical history for T2DM	49(19)	15(18)	34(19)	0.778^^^^
Medical history for heart diseases	16(6)	4(4.7)	12(7)	0.51^^^^
Medical history for chronic kidney diseases	19(7)	8(9.5)	11(6.2)	0.34^^^^
Medical history for HTN	70(27)	13(15)	57(32)	0.004^^^^
Medical history for foot ulcers	2(0.7)	-	2(1.1)	0.32^^^^
Do you smoke/ chew tobacco	31(12)	30(35)	1(0.5)	<0.01^^^^

** *Studying in grade 9 and 10 or have passed matriculation*

** *currently studying or completed education till class 6–8*

*** *above intermediate education*

! *including Balochi, Siraiki, Kashmiri*

+ *female respondents*

++ *include private job holders, government employees, marketing personnel, domestic aids, skilled and unskilled manual workers.*

^^ χ*2 test of independence*

^ *Mann-Whitney U test*

# *t-test for two independent samples*

Note: P-value employing χ2 test of independence for comparison of sociodemographic details of men and women, comes out to be < 0.01

Anthropometric and clinical assessments revealed a median BMI of 27.458 and 25.874 for men and women, respectively. 44% of participants were obese (BMI>=27 Kg/m2 as per Asian criteria-based categories adopted from [26]), and 45% of men and 83% of women had elevated waist circumferences (>=90 cm for men, >=80 cm for women). 16% of participants had a history of heart disease, 7% had chronic kidney disease, 19% had diabetes, and 27% had hypertension (15% among men vs. 32% among women, p:0.004). Tobacco use showed a significant gender disparity, with 35% of men and only 0.5% of women reporting use of chewing tobacco or cigarettes (p < 0.01).

### FINDRISC scoring

FINDRISC scores were calculated for the 211 participants who completed all eight questions and did not have a prior T2DM diagnosis (Table 2). While 66% of participants fell into the very low or low risk categories, one in four had high to very high-risk levels of developing T2DM in the next decade. On average, women had a significantly higher risk than men to develop T2DM. The risk of developing diabetes was positively associated with higher waist circumference and body mass index (BMI). Interestingly, the proportion of participants at the highest risk was greater among those categorized as overweight (BMI ≥ 23 & ≤ 26.9 kg/m²) compared to those categorized as obese (BMI ≥ 27 kg/m²). When comparing the full sample (n = 260) to the FINDRISC analysis sample (n = 211), we noted differential reductions across BMI categories after excluding diagnosed diabetes cases: 12.5% reduction in underweight, 19% in normal weight, 16% in overweight, and 21% in obese categories.

**Table 2:** FINDRISC scoring cross-tabulated with demographic variables.

S#	FINDRISC scoring (N = 211)	Very Low Risk (> 7) n (%) 64(30%)	Low Risk [78910–11] n (%) 75(36%)	Moderate Risk [1213–14] n (%) 20(9%)	High Risk [1516171819–20] n (%) 50(24%)	Very High Risk [2122232425–26] n (%) 2(1%)	p-value
**1**	**Gender**						<0.001^^^
	Male (69)	31 (45)	21 (30)	8 (12)	9 (13)	0 (0)	
	Female (142)	33 (23)	54 (38)	12 (8)	41 (29)	2 (1)	
2	**Age categories (Years)**						0.33^^^
	25 to 35 (106)	37 (35)	34 (32)	11 (10)	24 (23)	0 (0)	
	36 to 45 (61)	19 (31)	24 (39)	4 (7)	13 (21)	1 (2)	
	46 to 55 (30)	6 (20)	9 (30)	2 (7)	12 (40)	1 (3)	
	56 to 65 (14)	2 (14)	8 (57)	3 (21)	1 (7)	0 (0)	
**3**	**BMI categories**						0.04^^^
	Underweight (BMI < 18 Kg/m2)	5 (71)	2 (29)	0 (0)	0 (0)	0 (0)	
	Normal (BMI>=18 & <=22.9 Kg/m2)	17 (50)	7 (21)	3 (9)	7 (21)	0 (0)	
	Overweight (BMI>=23 & <=26.9 Kg/m2)	23 (29)	29 (36)	5 (6)	23 (29)	0 (0)	
	Obese (BMI>=27 Kg/m2)	19 (21)	37 (41)	12 (13)	20 (22)	2 (2)	
**4**	**Waist circumference**						0.00^^^
	Not Elevated Waist Circumference (Male<90 cm & Female<80 cm)	35 (56)	12 (19)	9 (14)	6 (10)	1 (2)	
	Elevated Waist Circumference (Male>=90 cm & Female>=80 cm)	29 (20)	63 (43)	11 (7)	44 (30)	1 (1)	
**5**	**Ethnicity**						0.01^^^
	Punjabi (106)	28 (26)	34 (32)	8 (8)	36 (34)	0 (0)	
	Pashtun (79)	23 (29)	34 (43)	9 (11)	11 (14)	2 (3)	
	Others (26)	13 (50)	7 (27)	3 (12)	3 (12)	0 (0)	
**6**	**Employment**						0.58^^^
	Unemployed (7)	2 (29)	3 (43)	1 (14)	1 (14)	0 (0)	
	Housewife (122)	32 (26)	46 (38)	9 (7)	33 (27)	2 (2)	
	Employed (82)	30 (37)	26 (32)	10 (12)	16 (20)	0 (0)	
**7**	**Education**						0.18^^^
	No Education (81)	23 (28)	35 (43)	11 (14)	10 (12)	2 (2)	
	Primary (34)	10 (29)	12 (35)	4 (12)	8 (24)	0 (0)	
	Secondary (68)	23 (34)	20 (29)	4 (6)	21 (31)	0 (0)	
	Higher (28)	8 (29)	8 (29)	1 (4)	11 (39)	0 (0)	
**8**	**Monthly Income (PKR)**						0.53^^^
	less than 11000 (6)	2 (33)	2 (33)	0 (0)	2 (33)	0 (0)	
	11000-20000 (91)	24 (26)	28 (31)	11 (12)	27 (30)	1 (1)	
	21000-30000 (62)	20 (32)	24 (39)	2 (3)	16 (26)	0 (0)	
	31000-40000 (20)	8 (40)	9 (45)	2 (10)	1 (5)	0 (0)	
	41000-50000 (25)	8 (32)	11 (44)	4 (16)	2 (8)	0 (0)	
	Greater than 50000 (7)	2 (29)	1 (14)	1 (14)	2 (29)	1 (14)	1 (14)
**9**	**Monthly Expenditure (PKR)**						0.68^^^
	less than 11000 (7)	2 (29)	2 (29)	0 (0)	3 (43)	0 (0)	
	11000-20000 (86)	21 (24)	27 (31)	11 (13)	26 (30)	1 (1)	
	21000-30000 (58)	19 (33)	23 (40)	2 (3)	14 (24)	0 (0)	
	31000-40000 (38)	16 (42)	14 (37)	3 (8)	5 (13)	0 (0)	
	41000-50000 (14)	2 (14)	7 (50)	4 (29)	1 (7)	0 (0)	
	Greater than 50000 (8)	4 (50)	2 (25)	0 (0)	1 (13)	1 (13)	

^ *Fisher’s Exact Test*

Nearly twice as many participants from the Punjabi rather than Pashtun ethnicity fell in the high or very high-risk categories (34% vs. 17%, p < 0.05). No statistically significant relationship was observed between employment status and risk scores. Risk increased with age until the oldest (56–65 years) bracket, where it declined. A U-shaped pattern was observed when examining risk by income level, with higher risk noted at both very low and very high-income levels.

The findings of FINDRISC scoring advocate that the novel tool is practical and can be effectively implemented in the real-world setting based on its simplicity yet comprehensive dimensional nature and easy integrational assessment without any additional pre-requisition. Additionally, the tool was practically feasible to implement in this setting, requiring minimal additional resources compared to standard HbA1c testing, especially in the low-income community of Dokh Hassu, Rawalpindi. No legal, regulatory, or ethical constraints limited the use of this tool.

### Assessment of knowledge about diabetes mellitus

Knowledge regarding symptoms, risk factors, and complications of diabetes was disaggregated by sex of the participants (Table 3). Awareness of ‘*clinical diagnosis of diabetes through blood tests’* was similar across sexes (male to female difference: 87% vs. 86%, *p*: 0.20). 21% of women and only 2.3% of men (p: < 0.01) reported knowing the difference between type I and type II diabetes, while 34% of women and 16% of men (*p*:0.01) responded that diabetes is a contagious disease.

**Table 3:** Knowledge and self-management behavior of participants regarding diabetes mellitus.

S#	Questions	Male n = 84	Female n = 176	*p-*value^!^
**1**	**Diabetes is clinically diagnosed by blood tests.**			0.20
	Agree	73(87)	151(86)	
	Disagree	–	6 (3)	
	Don’t know	11 (13)	19 (11)	
**2**	**A pregnant woman can have diabetes which usually disappears.**			0.03*
	Agree	31 (37)	94 (53)	
	Disagree	13 (15)	25 (14)	
	Don’t know	40 (47)	57 (32)	
**3**	**I know the difference between type-1 and type-2 DM.**			<0.01*
	Agree	2(2.3)	38 (21)	
	Disagree	82(98)	138(78)	
**5**	**Diabetes is contagious.**			0.01*
	Agree	14 (16)	60 (34)	
	Disagree	56(67)	92 (52)	
	Don’t know	14 (16)	24(13.6)	
**6**	**Diabetes is curable.**			0.70
	Agree	69(82)	150(85)	
	Disagree	7(8.3)	10(5.6)	
	Don’t know	8(9.5)	16 (9)	
***Knowledge Related to Symptoms of Diabetes***				
	**Following are the symptoms of Diabetes?**	**Male n = 84**	**Female n = 176**	***p-*value**
**i.**	**Increase thirst**			0.006*
	Agree	53(63)	143(81)	
	Disagree	8(9.5)	10(5.6)	
	Don’t know	23(27.3)	23 (13)	
**ii.**	**Frequent urination**			0.001*
	Agree	54(64)	148(84)	
	Disagree	9 (11)	6(3.4)	
	Don’t know	21 (25)	22 (12)	
**iii.**	**Extreme hunger**			0.002*
	Agree	48 (57)	137(78)	
	Disagree	9 (11)	8(4.5)	
	Don’t know	27 (32)	31 (17)	
**iv.**	**Unexplained weight loss**			0.001*
	Agree	43 (51)	131(74)	
	Disagree	7(8.3)	7 (4)	
	Don’t know	34(40.4)	38(21.5)	
**v.**	**Irritability**			<0.01*
	Agree	46 (58)	140(79)	
	Disagree	9 (11)	6(3.4)	
	Don’t know	29(34.5)	30 (17)	
**vi.**	**Blurred vision**			<0.01*
	Agree	45 (53)	135(77)	
	Disagree	8(9.5)	7 (4)	
	Don’t know	31 (37)	34(19.3)	
***Knowledge Related to Risk of Developing Diabetes***				
	**Risk of diabetes is higher for people with:**	**Male n = 84**	**Female n = 176**	***p-*value**
**i.**	**Family history of diabetes**			0.08
	Agree	45 (53)	113(64)	
	Disagree	18 (21)	20 (11)	
	Don’t know	21 (25)	43 (24)	
**ii.**	**Hypertension**			0.04*
	Agree	49 (58)	124(70)	
	Disagree	10 (12)	8(4.5)	
	Don’t know	25 (30)	44 (25)	
**iii.**	**Cholesterol**			0.06
	Agree	44 (52)	116(66)	
	Disagree	12 (14)	13(7.3)	
	Don’t know	28 (33)	47 (27)	
**iv.**	**Nicotine addiction**			<0.01
	Agree	26 (31)	103 (58)	
	Disagree	22 (26)	11(6.2)	
	Don’t know	36 (49)	62 (35)	
**v.**	**Obesity**			0.44
	Agree	54(64)	121(69)	
	Disagree	9 (11)	11(6.2)	
	Don’t know	21 (25)	44 (25)	
**vi.**	**Pregnancy**			
	Agree	23(27)	103(58)	<0.01
	Disagree	10(12)	8(4.5)	
	Don’t know	51(61)	65(37)	
***Knowledge related to complications of Diabetes***				
**i.**	**Long term complications from diabetes are easily reversible**			0.02*
	Agree	53(63)	137(78)	
	Disagree	17 (20)	16 (9)	
	Don’t know	14 (16)	23 (13)	
**ii.**	**An early-stage diagnosis of diabetes can help prevent long-term complications**			0.11
	Agree	67(78)	146(83)	
	Disagree	8(9.5)	6(3.4)	
	Don’t know	9 (11)	24(13.6)	
**c**	**In the long run, diabetes can lead to Blindness/ poor eyesight**			
**i.**	Agree	53(63)	127(72)	0.20
	Disagree	10 (12)	11(6.2)	
	Don’t know	21 (25)	38(21.5)	
**ii.**	**Heart problems**			0.03*
	Agree	49 (58)	129(73)	
	Disagree	11 (13)	11(6.2)	
	Don’t know	24(28.5)	36 (20)	
**iii.**	**Kidney failure**			0.002*
	Agree	46 (55)	123(70)	
	Disagree	15 (18)	9 (5)	
	Don’t know	23 (50)	44 (25)	
**iv.**	**Gangrene/ poor circulation/ amputation** Agree	48 (57) 15 (18)	131(74) 6(3.4)	<0.01*
	Disagree			
	Don’t know	21 (25)	39 (22)	
**v**	**Coma**			0.015*
	Agree	38 (45)	113(64)	
	Disagree	10 (12)	13 (7)	
	Don’t know	36 (49)	50 (28)	
	**Premature death**			0.03*
	Agree	49 (58)	130(74)	
	Disagree	7(8.3)	7 (4)	
	Don’t know	28(33.3)	39 (22)	
(B) Participants Self-Management Behavior Participants Regarding Diabetes Mellitus (n = 49)**				
	**Questions**	**Male n = 15**	**Female n = 34**	***p-*value**
**i.**	**Frequently do you go for diabetes checkup**			0.24
	Month or less	9(60)	9 (28)	
	Every two months	0(0)	3 (9)	
	Quarterly	0(0)	5 (15)	
	Bi-annually	0(0)	1 (3)	
	Annually	1 (7)	5 (15)	
	Whenever there is need	5 (33)	10 (30)	
**ii.**	**Made lifestyle changes after being diagnosed with diabetes**			0.44
	Yes	12(80)	30(88)	
	No	3 (20)	4 (12)	
**iii.**	**Monitor your glucose levels regularly**			0.04*
	Yes	6 (40)	24(70)	
	No	9(60)	10 (29)	
**iv.**	**Avoid or limit the consumption of any specific foods**			0.85
	Yes	12(80)	28(82)	
	No	3 (20)	6 (17)	
**v.**	**Take any medication to keep your glucose levels in check**			0.89
	Yes	13(86.7)	29(85)	
	No	2(13.3)	5 (15)	

! reporting p-values for χ2 test of association.

* *p-*value significance <=0.05

Regarding diabetes symptoms, women demonstrated greater awareness than men across multiple indicators: 81% of women versus 63% of men (p = 0.006) recognized increased thirst as a symptom; 74% of women versus 51% of men (p < 0.001) identified unexplained weight loss as a symptom. Similar patterns emerged for risk factor knowledge, with 70% of women versus 58% of men (p = 0.04) correctly identifying hypertension as a risk factor. For obesity as a risk factor, 69% of women and 64% of men showed agreement (p = 0.44).

Furthermore, knowledge of diabetes complications revealed that 78% of women versus 63% of men believed that ‘*long term complications of diabetes are easily reversible’* (*p*-0.02). Meanwhile, 73% of women and 58% of men (*p*:0.03) identified ‘*heart problems*’ as one of the long-term complications of diabetes.

### Self-management behaviors towards diabetes mellitus

Among the 49 participants with a previous clinical diagnosis of T2DM, self-management behaviors were assessed. Check-up visits and lifestyle changes did not differ by sex - 33% of men and 30% of women went for a check-up when they felt the need (Table 3). Similarly, dietary management was consistent across genders, with 80% of men and 82% of women reporting they avoided or limited certain foods to manage their diabetes. Most (80% of men and 88% of women, p: 0.44) had changed their lifestyle following a diagnosis. However, regular glucose monitoring was significantly more common among women than men (70% vs. 40%, p: 0.04) regularly monitored their glucose levels. Medication usage was high in both groups, with 87% of men and 85% of women reporting use of anti-diabetic medications.

### Cost of diabetes care

Among the 49 participants with clinically diagnosed T2DM, 63% had ever paid for their treatment, with no significant difference between men and women (67% vs. 62%, p: 0.74). The average expenditure was PKR 1747 for women compared to PKR 920 for men, but this difference was not statistically significant. Additionally, 40% of men and 28% of women had access to a glucometer, though this difference did not reach statistical significance (Table 4).

**Table 4:** Cost of diabetes (N = 49).

S#	Variables	Total	Male	Female
1	Do you pay for your diabetes treatment*	**N = 49**	**N = 15**	**N = 34**
	Yes	31(63)	10(67)	21(62)
	No	18 (37)	5 (33)	13 (38)
2	On average how much do you pay in a month (PKR)**	**N = 31**	**N = 10**	**N = 21**
	Oral medication	2774(2529)	1200(1000)	3524(2775)
	Insulin	1802(3219)	750(1359)	2304(3728)
	Insulin Syringes	1500(2895)	800(1857)	1833(3265)
	Glucose Strips	1024(1946)	305(505)	1367(2277)
3	**On average how much do you pay on every checkup/ doctor’s visit (PKR) ***	**N = 20** 1540(1059)	**N = 5** 920(602)	**N = 15** 1747(1111)
4	**Do you own a glucose meter** ^*^	**N = 49**	**N = 15**	**N = 34**
	Yes	19 (39)	6 (40)	13 (28)
	No	30(61)	9(60)	21(62)

* *Frequency is displayed with percentages in parenthesis for the categorical variables*

** *Means is displayed with standard deviation in parenthesis for the continuous variables usually given as Mean ± SD*

*** *PKR represents Pakistani Rupees*

## Discussion

Our study identified a notably high FINDRISC score among participants from low-income settlements, with 25% exhibiting elevated risks across both genders. Women faced twice the risk compared to men, underscoring the urgent need for targeted lifestyle modifications and aggressive preventive measures to manage and potentially prevent the development of T2DM, particularly among women.

Our study findings suggest that the FINDRISC tool is feasible for assessing future disease burden, particularly among women. The results indicate a higher burden compared to other regions of Pakistan and other South Asian countries [27]. Studies from South India and Bangladesh have reported high risks in 5% and 6% of their samples [28–29], respectively, while Tanzania and Southern Benin reported even lower proportions at 2% and 3% [30–31]. One clinic-based study from Karachi, Pakistan, showed a high risk among 7% of participants [27]. The markedly higher risk in our study likely reflects the greater prevalence of risk factors, particularly overweight and increased waist circumferences attributable to several factors prevalent in low-income settlements: physical space constraints in population-dense areas, lack of sidewalks and streetlights, socio-cultural restrictions on women’s mobility, and poor dietary practices in low-income settlements.

The urban environment of our study site significantly impacts physical activity levels. The community lacks nearby affordable or accessible public parks or exercise facilities, and homes provide insufficient space for physical activity. Studies on urban poverty, population density, and public health have consistently demonstrated that the built environment in inner cities profoundly affects physical activity levels, obesity rates, and overall health outcomes. [27,32–33]. While, the average BMI of Pakistan is 21.95 kg/m^2^ for men and 21.2 kg/m^2^ for women which is consistent with some of its neighboring countries such as India (21.9 kg/m^2^), Afghanistan (21.4 kg/m^2^), and Nepal (22.4 kg/m^2^) [34–35], and is much lower than the global average of 24.2 kg/m^2^ [36], our study was conducted in urban Punjab, which has both a higher BMI and a prevalence of diabetes and hypertension higher than the national average [37–38].

Our finding that overweight participants showed higher risk scores than obese participants on the FINDRISC assessment can be explained by examining the patterns of pre-existing diabetes diagnoses. The highest proportion of diagnosed diabetes was found in the obese category, meaning many high-risk obese individuals had already progressed to diabetes and were excluded from our risk assessment. This ‘depletion of susceptibles’ effect in the obese category underscores the importance of early intervention for overweight individuals before they progress to obesity and develop T2DM.

The observed sex disparities in diabetes management align with existing literature. [39–41]. This disparity may relate to the higher waist circumferences and limited physical activity among women. Housewives constituted 88% of women in our sample and typically experience limited physical activity due to restricted mobility and diminished agency within households [42–44]. Regional norms in South Asia restrict women’s physical activity to household chores or visiting neighbors, which can contribute to weight gain and increased diabetes risk [44–45]. A previous survey in our study location revealed that only 27% of women felt empowered to go to the lane store without a chaperone or permission [46]. Opportunities for walking and exercise in the inner city are further curtailed by congested living spaces, lack of public spaces for physical activity and cultural restrictions on women’s presence outside the home. In Dhok Hassu, where this study was conducted, 280,000 individuals live within 1.92 square kilometers, resulting in cramped individual residences and minimal walking requirements even for routine errands. Additionally, energy-dense local foods are commonly consumed in many Pakistani communities [47].

Spending on T2DM management is *PKR 1540 (USD 5.40)* per doctor’s visit, which is lower than the regional average. While women spend more in absolute terms on their care than men, this represents a smaller percentage of household income.

Previous research indicates that Pakistanis using both antihypertensive and antidiabetic medications typically spend approximately 6.5% to 7.81% of their household income on health [48], consistent with our findings of 8.5%. Within this, the proportional spending by men (12%) exceeds that of women (7%). Future research should explore potential contributing factors including disease severity, age differences, or socioeconomic disparities.

The study showed that implementation of the FINDRISC tool in a low-income urban community setting was feasible as a screening method for T2DM risk assessment. The questionnaire items are relatively straightforward and were easily understood by respondents. Data collectors recruited from the local community became proficient with the FINDRISC questions after just two days of training. This suggests potential for using the tool to screen high-risk individuals through basic outreach workers with limited education, an important first step toward identifying at-risk individuals and integrating them into counseling and treatment programs to help control the burden of T2DM and possibly hypertension. A key methodological insight was that the two-stage process resulted in higher dropout rates, indicating that anthropometric measurements should ideally be incorporated into the initial interview setting.

## Limitations

This pilot study, with its limited sample size, primarily aimed to assess the feasibility of using FINDRISC as a population screening tool rather than generating broadly generalizable findings. Since it was conducted in an urban informal settlement, the findings may not be representative of the broader Pakistani population of the country or even the provincial population. Additionally, the absence of FINDRISC data for 140 participants in the second (follow-up) phase was influenced by the timing of data collection. The household selection method may have introduced selection bias, particularly given that data collection occurred during daytime hours when many men were unavailable due to work commitments. This convenience sampling approach within households, where participants were selected based on availability and willingness to participate at the time of survey visit, may have affected the representativeness of our sample.“

An additional limitation is that several FINDRISC components rely on prior medical diagnoses or awareness (hypertension medication, history of high blood glucose, family history of diabetes). In our low-income settlement with limited healthcare access, these factors may be substantially underreported due to undiagnosed conditions and limited healthcare engagement. This likely results in underestimation of true diabetes risk, particularly among the most marginalized community members with the least healthcare access. Future adaptations of risk assessment tools for similar settings might consider modifications that rely less on previous medical diagnoses and more on directly observable risk factors. We relied on participants’ self-reported clinical diagnoses of T2DM without laboratory confirmation, which may introduce diagnostic uncertainty. Similarly, self-reported management practices are subject to recall and social desirability biases. Future studies should include verification through laboratory tests such as hemoglobin A1C, at least for a representative subset of participants.. However, some pre-diabetic individuals may present with normal test values, hence the utility of such testing may be limited if only identification of individuals at risk is intended. For comprehensive risk validation, longitudinal follow-up of respondents would be valuable to verify the predictive accuracy of risk assessments.

## Conclusion

The study demonstrated that FINDRISC could be feasibly implemented as a screening tool for T2DM risk assessment in this setting, using data collectors trained from the local community. Our study illustrates that resident of poor informal settlements, that make-up nearly 30% of Pakistan’s population, may face a higher risk for developing diabetes, with women particularly vulnerable. When individuals with diabetes seek treatment, the costs often represent a prohibitive proportion of household income, potentially deterring necessary care-seeking behaviors. The findings suggest potential value in wider community screening for T2DM risk, though longitudinal studies would be needed to confirm the predictive accuracy and cost-effectiveness of this approach in identifying individuals who go on to develop T2DM. Our study also highlights the public health importance of enhancing social-physical environments in urban-poor communities to reduce risks of T2DM and potentially other NCDs. Additional research is warranted, including scaled implementation of FINDRISC for risk assessment [27,32–33] and longitudinal follow-up of high-risk individuals to measure actualized outcomes and validate the screening approach in this context.

## Supporting information

S1 Table Name Description and Coding of Variables: Description and coding of all variables used in the FINDRISC scoring system and study analysis, including detailed scoring criteria for each component of the FINDRISC tool. (DOCX)

S1 Data Respondent anonymized raw data from the survey. (XLSX)
