# Supplementary material for: Sex differences in risk of developing Type 2 Diabetes Mellitus (T2DM): A feasibility assessment of FINDRISC scoring and barriers to disease management in a low-income settlement of Rawalpindi, Pakistan
Source: PLOS Glob Public Health. 2025 Jul 16;5(7):e0003087. doi: 10.1371/journal.pgph.0003087 (PMC12266448; doi:10.1371/journal.pgph.0003087)
Supplement: S1 Table — (DOCX) [file pgph.0003087.s001.docx]

**S1 Table: Name Description and Coding of Variables**

| **Variable Name** | **Description** | **Category** | **Score/Coding** |
| --- | --- | --- | --- |
| **FINDRISC** | | | |
| **Age** | Age variable depicting age through five categories | < 45 years  45–54 years  55–64 years  65+ years | 0 points  2 points  3 points  4 points |
| **BMI (kg/m^2^)** | Body mass index of participants categorized into three categories | <25 kg/m2  Between 25 and < 30 kg/m2   ≥30 kg/m2 | 0 points  1 point  3 points |
| **WC (cm)** | Categorical variable indicating waist circumference of the participants | Men: <94 cm; women: <80 cm  Men: 94–102 cm; women: 80–88 cm  Men: >102 cm; women: >88 cm | 0 points  3 points  4 points |
| **Physical activity (at least 30 min/day)** | Dummy variable if participants go physical activity | Yes  No | 0 points  2 points |
| **Fruit and vegetable intake** | Binary variable indicating whether participants have taken fruit and vegetables | Every day  Not every day | 0 points  1 point |
| **Regular medication for hypertension** | Binary variable if participants take regular medication for hypertension | Yes  No | 2 points  0 points |
| **History of high glucose levels** | Binary variable indicating whether participants have history of elevated glucose levels | Yes  No | 5 points  0 points |
| **Diabetes in relatives** | Categorical variable divided into three heads representing if participants had diabetes in relatives or not and to which degree | Yes, grandparents, cousins, uncle, aunt (2nd Degree)  Yes, parents, siblings, son, daughter (1st degree)  No | 3 points  5 points  0 points |
